# Progress in Social and Educational Inquiry Through Case Study: Generalization or Explanation?

**DOI:** 10.1007/s10615-016-0597-y

**Published:** 2016-07-23

**Authors:** Gary Thomas

**Affiliations:** University of Birmingham, Birmingham, B15 2TT UK

**Keywords:** Case study, Education, Generalization, Explanation

## Abstract

Although much of the most productive research in applied social science is case-based, there is still concern about the restricted utility of such research because of its limited power to offer generalizable findings. Such concern has contributed to a recent trend in policy-making circles—particularly those in education—to prefer experimentally orientated research for insights on policy. The argument is made here that concerns about generalization are exaggerated and that the focus upon them has allowed an evasion of issues about quality of explanation coming from different forms of social inquiry design. After discussing these generalization-based issues I proceed to define case study as an inquiry form, outlining its most significant ingredients and I offer a review of case study inquiries in education which exemplify its capacity for offering credible new insights on the questions being posed.

## Introduction

There have been many recent injunctions, including those from national governments, for researchers to use particular kinds of quantitatively orientated and experimental research in social and educational inquiry (see, for example, Goldacre 2013a, b; Prenzel 2009; Shavelson and Towne 2002; Slavin 2008; U.S. Department of Education 2004). Such research, it is sometimes asserted, provides “gold standard evidence.” I hope to make the case in this article, though, that the most influential, transformative education research comes not from the stable of experimental study but rather from explorations which are case orientated. Such research offers to education kinds of understanding which are inaccessible via formal kinds of trial and experiment. In using the “science of the singular” (Simons 1980) such inquiry promises to inform education practitioners in their own environments, where they can provide “research *in* practice, not research on practice,” as Friedman (2006, p. 132) has put it (see also, Cochran-Smith and Lytle 2009).

Over half a century and more, the most iconic analyses of education have come about from case study research, which can provide a uniquely vivid kind of inquiry and furnish the quality of analysis which is impossible from other kinds of research. Early examples include Philip Jackson’s (1968) *Life in Classrooms,* Harry Wolcott’s (1978) *The Man in the Principal’s Office*, Stephen Ball’s (1981) *Beachside Comprehensive* and Paul Willis’s (1993) *Learning to Labor,* all of which have contributed enormously to our understanding of the ways that schools work, teachers teach, and students learn. I shall look at these exemplars, and other examples of first-rate case study in education, later in this article.

While the case study has a relatively recent history in education, it has a longer pedigree in other disciplines. Garvin notes (2003) that it was a lawyer who had, in 1870, named case study method, with the use of the case study at that time in undergraduate teaching. The case had begun to be used, though, around the same time and a little before, in explicating and analyzing social and psychological phenomena. At the beginning of the nineteenth century, Jean-Marc-Gaspard Itard described his now-celebrated work with Victor, the “wild boy of Aveyron”, and later in the century Frédéric Le Play made his highly influential studies of the working and living conditions of French miners in the Jura (see Mogey 1955).

The aim of these early inquirers was to report and theorize about a particular person or set of people. Analysis based on this kind of work began to chime, at the beginning of the twentieth century, with new thought about social inquiry and how it should be undertaken. It resonated with new ideas about interpretative inquiry, encapsulated in the new anthropology and symbolic interactionism, in such a way that it became a force in and of itself. The case study, exemplified in, for example, Thomas and Znaniecki’s (1927/1958) explication of the life of American immigrants, *The Polish Peasant in Europe and America*, became an accepted and respected form of research.

Since then, the case study has been used increasingly to illuminate and explicate the social worlds we inhabit. And the different examples to which I have just been referring reveal very different kinds of case study with equally varied means of gathering data and analyzing it, from the use of people’s letters to each other, as in *The* *Polish Peasant …,* to rich, narrative accounts, as in Clifford Geertz’s (1973) notes on the Balinese cockfight. The fertility of the descriptions in these exemplars is sometimes quite striking—descriptions incorporating imagination, conjecture and theorization. The best case studies weave discussion and theorization with the presentation of the case account itself.

The case study presents a view of inquiry that takes a pragmatic view of knowledge—one that elevates a view of life in its complexity. It’s the realization that complexity in social affairs is often indivisible that has led to case study’s status as currently one of the most productive design frames open to the researcher. This is perhaps the reason behind its ongoing popularity among researchers in the field of education and other applied social sciences.

## What is Case Study?

There are strong commonalities about what case study constitutes across disciplinary boundaries. Reviewing a number of definitions of case study, Simons (2009) concludes that what unites them is a commitment to studying the complexity that is involved in real situations, and to defining case study other than by the methods of data collection that it employs. On the basis of these commonalities she offers this definition: “Case study is an in-depth exploration from multiple perspectives of the complexity and uniqueness of a particular project, policy, institution, program or system in a ‘real life’ context” (p. 21).

The emphasis in Simons’s definition is on depth of analysis. In it, one finds a “trade-off”, as Hammersley and Gomm (2000, p. 2) put it, between the rich, in-depth explanatory narrative emerging from a very restricted number of cases and the capacity for generalization that a larger sample of a wider population can offer. It is important to add to Simons’s definition the rider that case study should not be seen as a method in and of itself. Rather, it is a design frame that may incorporate a number of methods. Stake (2005) puts it thus:

Case study is not a methodological choice but a choice of what is to be studied … By whatever methods we choose to study *the case*. We could study it analytically or holistically, entirely by repeated measures or hermeneutically, organically or culturally, and by mixed methods—but we concentrate, at least for the time being, on the case. (p. 443)

Choice of method, then, does not define case study: analytical eclecticism in the in-depth study of a subject of interest is the key. Alongside holism and methodological eclecticism the case inquirer needs carefully to consider the nature of what is being studied, analytically speaking.

As I have discussed elsewhere (Thomas 2013), case study is one of the scaffolds that can help to structure the design of research. As I have defined them (Thomas 2011) case studies are

… analyses of persons, events, decisions, periods, projects, policies, institutions or other systems which are studied holistically by one or more methods. The case that is the subject of the inquiry will be an instance of a class of phenomena that provides an analytical frame—an object—within which the study is conducted and which the case illuminates and explicates.

The emphasis in this definition is on analysis; I try to make it clear that while case inquiry may often rely on observation, and to an extent description, these are not ends in themselves and the best case studies go much further than illumination. The definition makes a separation between the subject, the focus, of the study and the theoretical issue that this subject explicates. In it I have drawn on the work of Wieviorka (1992), who made the point that a case in a case study cannot be *simply* an instance of a class. Wieviorka unpacked in more detail the distinctions between the case and the class by noting that when we talk about a case we are in fact talking about two elements: first, there is what he calls a ‘practical, historical unity’ (p. 159). We might call this *the subject*. Second, there is what he calls the ‘theoretical, scientific basis’ of the case.

In other words, it is important for case inquirers to be clear about what the case study is a case study *of.* A case study, as a *study* (as distinct from a case illustration or a case history) must in some sense explicate a wider theme: it must help in our understanding of some theoretical issue.

## Methodological Issues for the Case Study in Education

### Generalization

… situations are so varied that even a large number of cases may be a misleading sample … and none is comprehensible outside the historical sequence in which it grew.

(Vickers 1965, p. 173)

Here, Vickers states the principal reason for the sometimes suspect status of case study as a research design form. This suspicion stems principally from the assumed paucity of general understanding offered by case study. It is *general* understanding that is the key, and *generality* goes to the heart of the matter, for it is here, in generality or universals that we find issues of what social science, and particularly theory in social science, has distinctively to offer. This emphasis on generalized knowledge is a problem for case study, which appears to offer little in the way of generalizable information to social scientific inquiries.

Bassey (2001), however, writing from the context of education, notes that “it is possible to distinguish between two modes of research, namely search for generalities and study of singularities” (p. 6). He picks up Simons’s (1980) notion of the “singularity” of the educational situation—that singular status implying everything within the boundary of what is under study. It is, as Bassey puts it (ibid), “one set of circumstances and the events, people, places and things, which constitute that set of circumstances, [which] are treated in the study as an entity.”

Bassey firmly sets the issue of generalization in the context of the classroom. He says:

Open generalizations give reliable predictions and so are obviously valuable in the making of classroom decisions. But, in my view, they are scarce in number and so once these few have been mastered, and have become an integral part of a teacher’s way of operating, they appear obvious and no longer valuable.

He concludes that the education research community should

… distinguish between pedagogic research and other forms of educational research, and in relation to pedagogic research should eschew the pursuit of generalizations, unless their potential usefulness is apparent, and instead should actively encourage the descriptive and evaluative study of single pedagogic events.

I have continued the discussion about generalization elsewhere (Thomas 2011), noting that “the study must be framed not in the diluted constructs of generalizing natural science but rather in questioning and surprise, heuristic, particularity, analogy, consonance or dissonance with my own situation” (p. 33). The case study, I have concluded, is of course about understanding some phenomenon or construct, but understanding it in the context of what Gadamer (1975) calls one’s “horizon of meaning” (p. 269). The conclusion is that while precise forms of generalization are impossible—particularly the tight generalization of the natural scientist—the obverse of this observation is that no situation is unique: each is interpreted in the context of our own experience. To interpret in the context of one’s own experience is both legitimate and valid.

For me, the issue about generalization is less troublesome than many fear, for much scientific inquiry is not actually about generalization but, rather, understanding. This is true in any domain of inquiry. Scientists—from astronomers to zoologists—seek understandings on the basis of evidence, which is painstakingly sought, evaluated and used to make the best possible conjectures and explanations of the phenomena in question. While some of these explanations will require certain kinds of rigorous generalization, others do not.

## Is It Science?

Atkinson and Delamont (1985) argue trenchantly for the need for case inquirers to develop a well formulated body of theory and methods in order to produce a coherent, cumulative research tradition. In doing this, they are developing a theme that has been much discussed in qualitative research. The issue is about science and legitimacy of this or that method (Thomas and James 2006) and here Kemmis (1980) makes the point that case studies are sometimes dismissed as purely subjective. They are thus seen as unscientific and are regarded with suspicion, even hostility, by some social scientists. He makes the point that case study is indeed science: it is truth-seeking and in the quest for public knowledge. In discussing the putative pillars of scientific credibility in social science—reliability and validity—he asks what estimates of reliability can be given for a field-note jotted down in the chaos of a classroom discussion.

Lather (2004) also takes on the theme of science, regretting the call for certain *kinds* of science in recent government reports—particularly in the discourse which stems from the landmark US piece of education legislation, *No Child Left Behind*, which demanded that teachers use only scientifically proven methods in their teaching. It’s a theme I have taken up myself (Thomas 2012): the point is that there is no core to scientific method, no charmed circle of precepts and processes that lead the incipiently scientific inquirer to the sunlit uplands of scientific inquiry. My argument, similar to Lather’s, is about the ways that we choose to be scientific in education inquiry and the consequences that such choices have for the nature and growth of our field of endeavor—our own science.

Stenhouse (1978, 1980) conjoins discussion of these issues that concern the legitimacy of case study with concern about generalization. He sets case study in the context of research and what research should be. He is concerned in particular about verification and cumulation in case studies conducted in field settings in education, and he concludes that case study is a basis for generalization and hence cumulation of data. He proceeds to assert, in response to questions about the usefulness of case study that practice will improve when experience is systematically marshalled as history. He asks for the accumulation of an archive of case records. The concern is to provide a cumulative body of knowledge. But, as I have suggested elsewhere (Thomas 2012) expectation about cumulation in our scientific inquiry in education has to rest on an accumulation not of generalizable facts but of *understandings* drawn from and assessed in the context of one’s own experiences and the experiences of others. It rests, in other words, in the cultivation of provisional, tentative models for interpretation and analysis.

Smith (1978) described well the process of cultivating tentative models for interpretation in his account of the “miniature theories” (p. 363) which teachers develop and share (and see also Cochran-Smith and Lytle 2009). Ideas about how it can be conducted have traveled various avenues from Lewin’s (1946) action research to Checkland’s soft systems (1981) to Bryk et al’s (2015) improvement science.

## Some Examples of Case Study in Educational Science

I have already mentioned three classic texts—Paul Willis’s *Learning to Labor,* Harry Wolcott’s *The Man in the Principal’s Office* and Stephen Ball’s *Beachside Comprehensive*—and it is worth going into some more detail on these before looking at other exemplars of the case study design frame.

Using case study, each of these researchers has done much for our understanding of the ways that schools work. They have achieved this by painting pictures in fine-grain detail about the encounters that occur in schools amongst staff and students.

*Learning to Labour* is often described as a classic ethnography. In it, Willis untangles how the young people at the “Hammertown” school—a school with a predominantly working class catchment in the English midlands—developed an antagonism towards school. They developed what Willis calls a *counter school culture*. They did this via what Willis calls *differentiation*. He says “*Differentiation* is the process whereby the typical exchanges expected in the formal institutional paradigm are reinterpreted, separated and discriminated with respect to working class interests, feelings and meanings” (p. 62). He intertwines the development of the theoretical narrative about differentiation and counter culture with observations and illustrations from the case study itself. There is surely no way that such insights could have come from any frame of research other than case study here.

Ball (1981), in *Beachside Comprehensive*, presents a case study of a school and its pupils at a particular moment of change for education. He seeks to understand how the pupils “make sense of school as part of their whole life-world” (p. 109). His work is interesting as case study for the data-collection methods that he uses (questionnaires, diaries) and the ways that he simultaneously incorporates insights from the work of others. In an echo of the “differentiation” and “counter culture” of Willis, Ball reveals how, especially in the final year of compulsory education at a time when the school leaving age was rising, pupils accepted or rejected the goals of the school, and how those who more conspicuously rejected it were in turn viewed as failures by the teachers.

Before both of these studies, in 1973, was Wolcott’s *The Man in the Principal’s Office: an Ethnography,* which was one of the first detailed ethnographies undertaken in education. The work shows the range of data collection and analytical techniques open to the case inquirer. Wolcott notes the contradiction present in educators’ espoused wish to be seen as integrated with their communities while making their own subculture at school a relatively closed one.

Then there are case studies which reveal their power to change through enabling genuinely fresh theoretical insight. From the very beginning of Ferguson’s (1992) *The Puzzle of Inclusion: a Case Study of Autistic Students in the Life of One High School* the reader is immersed in the case. Immediately, we are encouraged to think about the situation itself, to hypothesize, to make our own assessments and judgements about what is happening. The author, therefore, relinquishes control over the interpretations, as Sparkes (2007) puts it—interpretations about the integration of autistic students into a mainstream school. The case is fascinating for the insights it offers on inclusion. Importantly for case study, Ferguson challenges any assumption that his case study school is in any way typical, nor need it be, he says. He concludes with a key statement:

Each high school … has its own set of unique events and specific personalities that interact with larger social forces and structures to construct its own pattern of understanding itself. Case studies are intended to reveal those patterns in as rich detail as possible. This does not mean that generalizations are impossible or even undesirable. Rather it simply places most of the responsibility for generalization to other settings on the readers themselves who know those other settings best. It is my responsibility as the writer to provide a thick enough description for the readers to make such judgments and comparisons. (p. 166)

Ferguson vividly illuminates the work of the case inquirer here. It reminds us that the work of the researcher in this form is truly theoretically grounded, with the constructs emerging from the research itself rather than being orphaned to some preordained theoretical construct.

In this, Ferguson’s work is like Wright’s (2010) case study of a small child and her mother. This chronicles, reflects upon and analyzes the emotional stasis and eventual thawing and trust of a little girl with whom Wright was working. Because of the case study approach, it is refreshingly free of the quasi-explanatory constructs that so often characterize accounts of breakdown in learning or emotional development at school. Wright’s explanation about the girl’s withdrawal comes directly from what he saw and what he knew. His intuitions about how to behave with her came from his own experiences as a person and a professional, one with experience of other people, and one who approaches others with humanity, understanding and a will to succeed. We, the readers, read in the context of our own experience, our own horizons of understanding.

In a study of reading failure, Johnston (1985) did something similar. He gave a case study examination of reading failure and found reasons for this failure more in students’ anxiety than in putative psychological deficits, where traditional educational and psychological science so often have sought within-child explanations. Like Ferguson and Wright, Johnston found failure at school to depend on the context and culture for learning. It is only through the rich and detailed study of individual cases that such analyses of children’s difficulties at school can be made. Such work shows that students’ success or failure at school is due less to “learning disabilities” and more to an array of factors around which acceptance and inclusion are constructed.

A similar set of new, rich explanations, divorced from the traditional starting points of the educator looking for explanation of why children fail come from Hart et al. (2004), who tell the story of one teacher, Julie. It’s one case study among nine in their book *Learning without Limits*, describing and analyzing how teachers developed alternative practices in their classrooms to move away from notions of fixed ability and disability, including “learning disability”. It shows how teachers use principles of “accessibility” and “emotional well-being” together with expectations about minimum levels of achievement for each child. Hart and her colleagues are putting into practice what Ferguson was suggesting—enabling through rich description an assessment by the reader of the transferability from one situation to another.

All of these case studies force serious re-thought about many of the pseudo-scientific constructs around which “failure” at school is often constructed. They do this by compelling a direct analysis of the case that is in front of the inquirer. The analyses come not from pre-packaged theorization that puts “failure” into this or that box with this or that label, but rather from insights which emerge from the authors’ own experiences as people and as professionals. We read their accounts and understand them in the contexts of our own experiences, our own horizons of understanding.

There are other examples of case study use in education that demonstrate well that this form of design need not follow an ethnographic route. Cremin et al. (2005) outline the use of what is sometimes called an n = 1 design, unusual in the case study genre for its employment of an experimental approach. As I have noted, methodologists such as Stake (2005; 443) have emphasized that case study is not a methodological choice but rather “a choice of what is to be studied” and Cremin et al. demonstrate this point in this experimental study. The researchers look at six classrooms in detail, examining the work of teaching assistants and in particular imposing three different kinds of organization for the work of those assistants in the classrooms. The different organizational methods are compared using a repeated measures experimental design and the findings of this are complemented by commentary from the staff participating in the study.

The terms “experiment” and “case study” are also juxtaposed by Driessen and Pyfer (1975), though here the “experiment” is an experiment only in the sense of trying something out. These researchers report on an evaluation of a program in adult basic education which was given in informal home settings instead of traditional classrooms. The aim was to meet the needs of “208 adults who wanted formal educational skills, but who found it neither comfortable nor appealing to participate in formal classroom settings” (p. 112). The whole trial is analyzed qualitatively. The use of the term “experiment” in this kind of study raises the issue of what a scientific experiment needs to look like in social science. It needn’t look like the experiments used in plant science and medicine. It can be far simpler, and I have discussed elsewhere the expropriation of the term “experiment” (Thomas 2016).

García et al. (2012) give literally a case *history* of the Oxnard schools in California—a history of what the authors call “mundane racism” (p. 2)—almost routine, taken-for-granted racism. Using school records and census records, they show how the school board’s decade-long “obsession” (p. 2) with segregation “effectively established a permanent dual schooling system that replicated racial hierarchy” (p. 2). This ingenious work both motivates and informs, providing not just a window on practices formative of some of today’s prejudices but also insights about how to move forward.

Two further examples demonstrate the value of the case study approach in education in the whole process of understanding teaching, learning and development. Duckworth (1986) reflects on a project in which she as a researcher and tutor asked teachers to engage in moon-watching—as a novel kind of task, the kind wherein empathy could be experienced with classroom learners—in order to reflect on their understanding of the sort of learning and teaching that might be expected at school. She concludes that “they make sense by trying out their own ideas, by explaining what they think and why, and seeing how this holds up in other people’s eyes, in their own eyes, and in the light of the phenomena they are trying to understand” (p. 487). This is summed up in the understanding of “teaching as research”. Hennessy, Mercer and Warwick do something similar (2011), showing how researchers and teachers could co-construct this process. They describe co-inquiry wherein, as the authors put it, “collaborative theory-building” (p. 1910) happened. Out of the process, pedagogical rationales were shifted and altered. The authors describe the ways that the case study enabled elucidatory work with teachers, suggesting that a rich set of perspectives could emerge: all the teachers would discuss insights which might develop as they orientated themselves to others’ perspectives.

I’ll finish this mini-sample of case studies in education with Jiménez and Gersten’s (1999) analysis of two distinct approaches to instruction provided by two bilingual teachers. They offer these as lessons and dilemmas which they have drawn from the literacy instruction of these two teachers. Their conclusion about the “method” of their work sums up much of the method of the case inquirer, for they say that their work aims to create what Wolcott called *little theories.* They note that Wolcott believed that education is best served by the generation of multiple insights tailored to specific situations and grounded in the expertise of those who work in those situations. These little theories, they suggest, are inductively derived conclusions concerning instruction and learning.

## Conclusions

Case study is about explanation through in-depth inquiry and insider accounts, producing “little theories” and “miniature theories”, via the “multiple realities” of Berger and Luckmann (1979). These prove to be the life-blood of serious, transformative inquiry in education. All of the studies I have drawn from in the previous section of this article force serious re-thought about many of the pseudo-scientific constructs around which ideas about students’ experience at school is often constructed. They do this by compelling a direct analysis of the case that is in front of the inquirer. The analyses come not from the kind of pre-packaged theorization which so often guides the understanding of putatively gold standard experimentation, but rather from insights which emerge from the authors’ own experiences as people and as professionals.

In whatever field, scientific inquiry seeks to answer questions and to solve puzzles. That is its purpose. It looks for explanations—clarification, illumination, enlightenment—about how and why things happen as they do. We conjoin ideas, make connections, test hypotheses, recognize themes, and build models of the way the world works. We seek, as Einstein put it, “in whatever manner is suitable, a simplified and lucid image of the world” (cited in Holton 1995, p. 168). Our inquiries, our questions and answers, assist in building what Harré (2012)—in explaining the purpose of social science—called “working models of some aspect of social life.” We do this eclectically, and we do it, natural scientists and social scientists alike, through case study as much as experimentation.

For social scientists also seek “a simplified and lucid image” of the worlds in which they work—in whatever way. There can be no specific, superior type of question; no sunlit path to the perfect inquiry. Rather, there is variety. But this variety should not be seen as social science’s Achilles’ heel, accompanied by a laying out of hierarchies of better and worse kinds of research. We should, cherish, not disown, methodological pluralism and value the insights and understandings which come from case study.

None of this, of course—none of the call for pluralism and complementarity, with appropriate respect afforded to case study, ethnographic or more generally qualitative social inquiry forms—is to deny the absolute need for rigor in the conduct and analysis of research. As sociologist Robert Merton (1976) argued some time ago, the need is for “disciplined eclecticism” (p. 169) and his entreaty is still relevant. Funders need to be convinced of the quality and the intermeshing contributions of different forms of inquiry. They need to be convinced, in other words, of the matrix-like nature of inquiry forms, as Hammersley (2015) put it. Many advocates of experimental methodology recognize this—recognize, in other words, the slenderness of insight provided by experimental work and incorporate case study and other qualitative elements into their design frameworks to provide such insights. The onus has to be on case inquirers to argue for the contribution of idiographic inquiry to the findings of such research, as well arguing for the analytic power demonstrated in the kinds of study I have reviewed in this paper.
